# Stature estimation from handprint measurements: an application to the medicolegal investigation

**DOI:** 10.1186/s41935-020-00215-1

**Published:** 2021-01-07

**Authors:** Md. Asadujjaman, Md. Golzer Hossain, Md. Sohel Rana, Md. Zahidul Islam

**Affiliations:** 1 Department of Industrial & Production Engineering, Rajshahi University of Engineering & Technology, Rajshahi, Bangladesh; 2 School of Engineering & Information Technology, University of New South Wales, Canberra, Australia

**Keywords:** Stature estimation, Handprint measurements, Forensic anthropmetry, Forensic identification, Medicolegal investigation, Regression analysis

## Abstract

**Background:**

The estimation of the stature of human beings is a major part of medicolegal investigation when only body parts are found. The study aimed to estimate the stature from different handprint measurements in a Bangladeshi population using statistical considerations. A sample of 200 young Bangladeshi adults (100 men and 100 women) with no physical disabilities participated in this study. Stature and seven anthropometric measurements were measured using standard anthropometric measurements. The bilateral asymmetry was tested using the independent *t* test. The Pearson’s correlation coefficient (*R*) between the stature and different handprint measurements was calculated. Consequently, the simple and multiple linear regression models were developed to estimate the stature from the handprint measurements.

**Results:**

The bilateral asymmetry was statistically not significant (*p* > 0.05) in right and left handprints. Sex difference significantly (*p* < 0.05) influences the relationship between stature and handprint measurements. A positive and strong coefficient of correlation (*R*) value presents between stature and the handprint measurements. The right 2^nd^ digit length in men (*R* = + 0.709, *R*^*2*^ = 0.502, SEE = ± 44.141 mm) and the right handprint length in women (*R* = + 0.552, *R*^2^ = 0.305, SEE = ± 49.074 mm) were the most reliable estimator of stature. However, when data were combined for both sexes, the right handprint length was identified as the most reliable estimator of stature with higher values of *R* (+ 0.777) and *R*^2^ (0.603), and a lower value of *SEE* (± 55.520). Multiple regression equation showed greater reliability than linear regression equations in stature estimation from handprint measurements in Bangladeshi population.

**Conclusions:**

It was concluded that the estimation of stature from handprint measurements is possible and reliable. The findings of this study are very useful from the forensic and medicolegal point of view and can use to estimate the stature in Bangladeshi population.

## Background

The identification of the criminal or the perpetrator is the primary goal of a case solver to deal with a crime case. This process can be hindered by lack of evidence found at the crime scene. However, it would be a great support for the law enforcement agency if a biological profile of the suspects can be estimated and developed from the scarce evidence. It would help them in narrowing down the pool of suspects.

Stature is considered as one of the “Big Four” parameters for the development of the biological profile (Ahemad and Purkait 2011; Krogman 1955; Paulis 2015). A lot of research work has been conducted in the past for estimating the stature form numerous body dimensions; example includes the dimensions of the head (Pelin et al. 2010), hand (Asadujjaman et al. 2019; Ishak et al. 2012b; Kalia et al. 2008; Kim et al. 2018; Krishan and Sharma 2007; Uhrová et al. 2015; Zulkifly et al. 2018), foot (Asadujjaman et al. 2020a; Hisham et al. 2012; Kanchan et al. 2008, 2010; Krishan et al. 2011, 2012; Kuan 2018; Ozden et al. 2005; Sanli et al. 2005; Uhrová et al. 2013, 2015; Zeybek et al. 2008), lower limb (Ahmed 2013a; Nor et al. 2013), upper limb (Ahmed 2013b; Ismail et al. 2018), cranium (Shrestha et al. 2015), sternum (Yonguc et al. 2015), lumber vertebrae (Zhang et al. 2015), femur and tibia (Brits et al. 2017), etc. Apart from these, handprints (Ahemad and Purkait 2011; Ishak et al. 2012a, b; Krishan et al. 2015; Moorthy and Yin 2016; Paulis 2015; Salama 2013; Zulkifly et al. 2018) and footprints (Abledu et al. 2016; Asadujjaman et al. 2020b; Caplova et al. 2018; Fawzy and Kamal 2010; Kanchan et al. 2012; Khan and Nataraja Moorthy 2015; Krishan 2008; Robbins 1986) have already been used in various populations for estimating the stature as these are one of the commonly available clues found at the crime scene.

Although numerous studies have been conducted for estimating the stature from footprints notwithstanding less work has performed on the handprints. In the past studies, it has been found that the handprint dimensions can be successfully used for the estimation of the stature (Ishak et al. 2012b; Krishan et al. 2015; Moorthy and Yin 2016; Paulis 2015; Salama 2013; Zulkifly et al. 2018). Those studies revealed a strong positive correlation between handprint dimensions and stature for both hands and sexes. Thus, simple (Ahemad and Purkait 2011; Ishak et al. 2012b; Paulis 2015; Zulkifly et al. 2018) and multiple linear regression models (Ishak et al. 2012b; Paulis 2015; Zulkifly et al. 2018) were successfully used by previous researchers for estimating the stature. The study of Ahemad and Purkait (2011) on 503 Indian men revealed the strong positive correlation (*p* < 0.05) between handprint dimensions and stature. Among the seventeen hand dimensions studied, hand length was the most reliable parameter for estimating the stature. Ishak et al. (2012b) analyzed seven handprint dimensions in a Western Australian population and found a strong positive correlation (*p* < 0.01) between handprint dimensions and stature in both sexes. Moreover, Paulis (2015) conducted a study on the Egyptian population and used simple and multiple linear regression for estimation stature from handprint dimensions. The study revealed that hand length was the most reliable single factor for estimation stature from handprint dimensions. Similarly, Zulkifly et al. (2018) found a strong positive correlation (*p* < 0.05) between handprint dimensions and stature on Iban subjects in both sexes. Zulkifly et al. (2018) reported the handprint length as the most reliable estimator of stature; the correlation coefficient between stature and handprint length was ranged between + 0.59 and + 0.68.

Anthropometric measurements vary from one population to another (Asadujjaman et al. 2017, 2020b). The degree of access to nutrition and health services may have an effect on the stature of the different populations around the world (Perkins et al. 2016). Moreover, as genetics can play a vital role in human growth, therefore the previous studies based on a particular population cannot be used for estimating stature from handprints for other populations.

In Bangladesh, to date, to the best of our knowledge, no study has conducted to estimate the stature from handprint measurements in this population. Therefore, this study aimed to investigate the possibility of establishing the relationship between stature and handprints, and finally to derive standard linear and multiple regression formula for the Bangladeshi population to estimate the stature from handprint measurements.

## Methods

### Materials

In this study, data were obtained from 200 volunteers having no physical disability (100 male and 100 female) aging from 18 to 30 years (23.62 ± 2.96 years in males and 23.58 ± 3.48 years in females) old. A separate questionnaire for collecting basic demographic information such as sex and age was provided while collecting the measurements of stature and handprints of each subject. Consent and permission for using their personal data for research work were collected from the volunteers in written form. The researchers are committed to protecting the privacy of personal information of the participants.

### Handprint acquisition

**Fig. 1:**
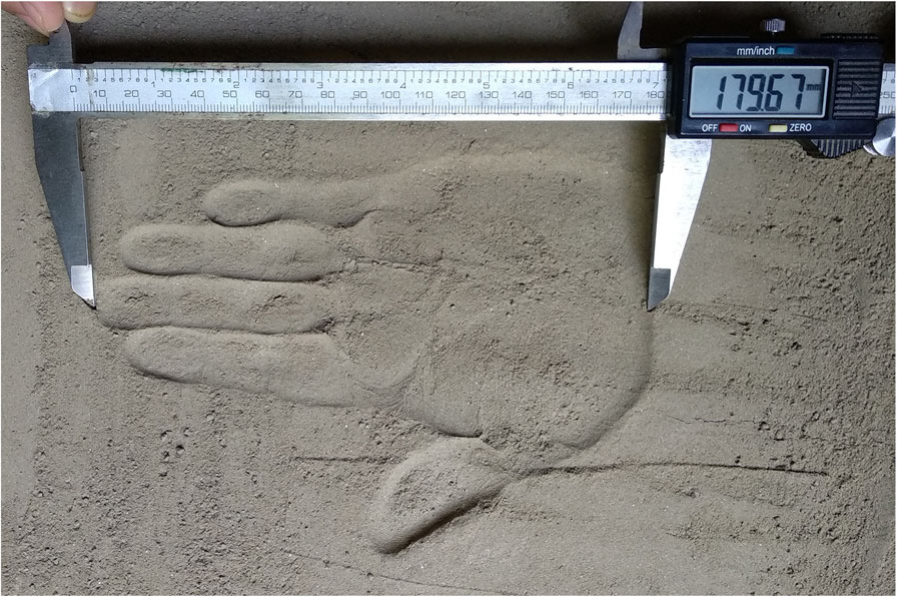
Technique of taking 3^rd^ digit length or hand length from a handprint

A flat box with powdered soil was used for collecting the handprints of both hands of the subjects. The subject was asked to make hand impressions on the powdered soil and thus, a digital slide calliper was used for acquiring the measurement of handprints. While taking the hand impression on the powdered soil, it was ensured that the powdered soil was flat every time. The technique to measure the hand length from handprint is shown in Fig. 1.

### Measurements

**Fig. 2:**
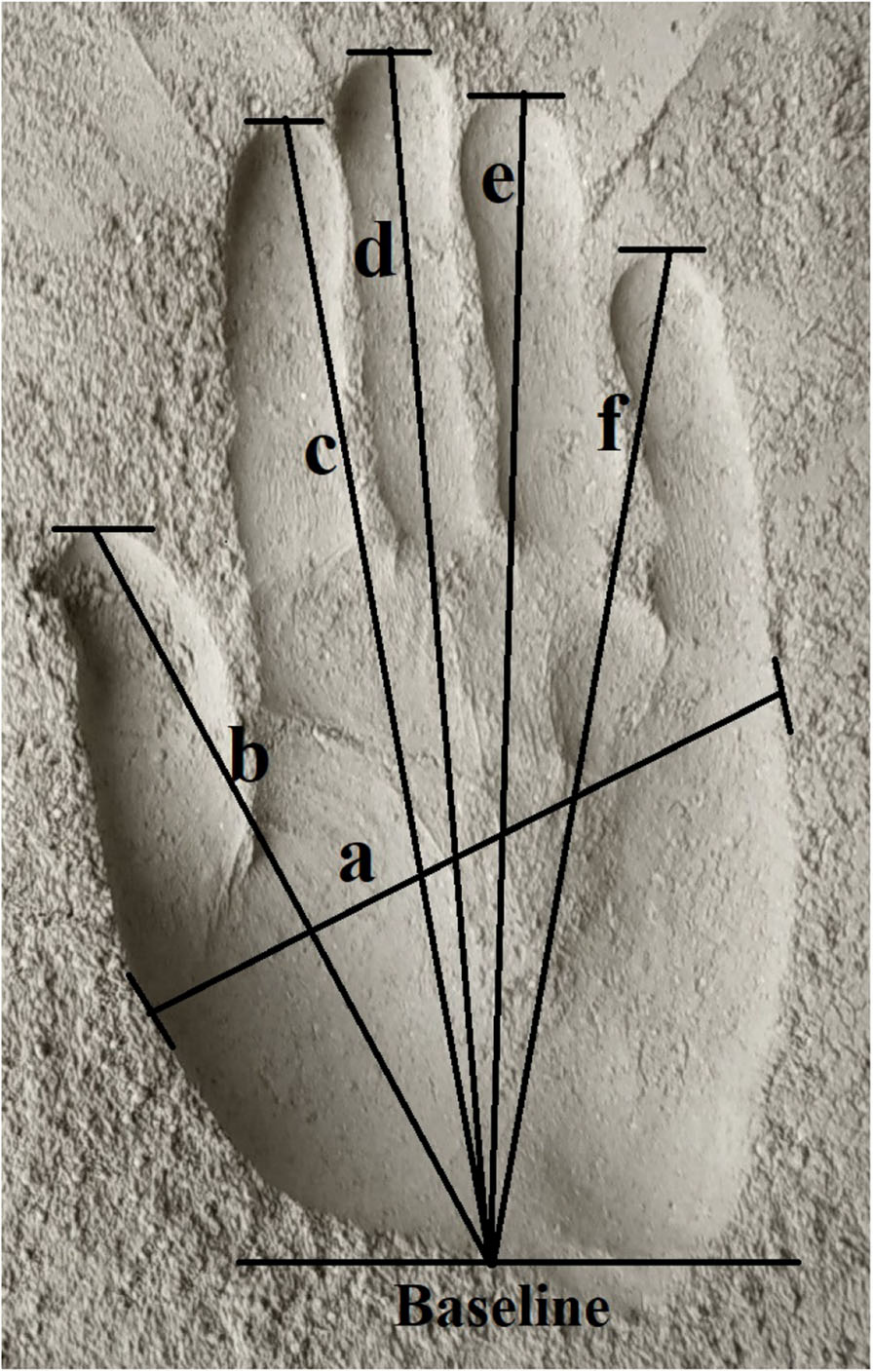
Handprint measurements: **a** maximum handprint breadth; **b** 1^st^ digit length; **c** 2^nd^ digit length; **d** 3^rd^ digit length; **e** 4^th^ digit length; **f** 5^th^ digit length

Stature and seven handprint measurements (handprint breadth, maximum handprint breadth, 1^st^ digit length, 2^nd^ digit length, 3^rd^ digit length, 4^th^ digit length, and 5^th^ digit length) were measured using standard measuring techniques. The anthropometric measurements were followed by the study of Moorthy and Yin (2016). Measurements of handprint were taken for both left and right hands. Different handprint measurements are illustrated in Fig. 2. All the studied handprint measurements were taken from the baseline shown in Fig. 2.

Stature is the person’s natural height in an upright position. The subject was asked to stand in an erect position without any wear on foot and head to take the measurement. Therefore, the distance from the floor to the highest point of the head was taken as the stature. The maximum handprint breadth is the distance from the most lateral point of the thumb finger metacarpal head to the most medial point of the little finger metacarpal head with closing the fingers of the handprint. The 1^st^ 2^nd^, 3^rd^, 4^th^, and 5^th^ digit lengths are the distance between the midpoints of the distal transverse crease of the wrist to the thumb, index, middle, ring, and little fingertip of the handprint, respectively. The handprint length is the 3^rd^ digit length of the handprint.

To avoid inter-observer error, all the measurements were taken by one observer. To limit measurement error, all the measurements of each subject was taken twice, and the mean value was taken. If two primary measurements have a difference of more than 4 mm, then both data were rejected, and again two new measurements were taken, and the mean was used. Intra-observer measurement errors for 30 subjects were then calculated. The measurement margin error, then calculated using the absolute technical error measurement (TEM), relative technical error measurement (rTEM), and coefficient of reliability (*r*) (Kim et al. 2018; Ulijaszek and Kerr 1999).

### Statistical analysis

For statistical analysis of the data, SPSS statistical software (version 23.0) and Microsoft Excel 13 was used. Descriptive statistics such as mean and standard deviation were calculated. A paired sample *t* test was performed to analyze the bilateral asymmetry in handprint measurement. An analysis of covariance using the general linear model (GLM) was conducted to test whether sex has an effect on the stature and handprint measurements. To estimate the stature from handprint measurements, simple linear and multiple linear regression models were used. Pearson correlation coefficient (*R*) was used for establishing the relationship between stature and handprint measurements. *R* value indicates the strength and direction of the relationship between the stature and handprint measurements. The coefficient of determination (*R*^2^), standard error of estimation (SSE), and *p* values were also used to describe the prediction of stature from handprint measurements from regression equations. *R*^2^ interprets the proportion of the variance in stature that is estimated from the handprint dimensions. The values of *R*^2^ vary from 0 to 1. Higher values of *R* and *R*^2^ means a greater reliability in predicting the stature from handprint measurements with a lower prediction error. The SEE predicts the deviation of estimated stature from the actual value. It represents the average distance that the observed stature measurement falls from the regression line. A low value of SEE means greater reliability in the stature estimation. *P* value shows the statistical significance in stature estimation from the handprint measurements. A *p* value less than 0.05 is statistically significant.

## Results

Table 1 shows the assessment of the intra-observer error in the measurement variables used in this study. In measurement variables, rTEM was less than 5% and the *r* values were higher than 0.97 in measurement variables. According to Ulijaszek and Kerr (1999), intra-observer error was regarded as an acceptable range for the measurement variables.

**Table 1:** Intra-observer error measurement

Measurement	Absolute TEM	rTEM%	***r***
Right MHB	0.635	0.582	0.973
Left MHB	0.620	0.574	0.974
Right F1DL	0.657	0.537	0.991
Left F1DL	0.617	0.508	0.993
Right F2DL	0.650	0.638	0.993
Left F2DL	0.602	0.343	0.994
Right F3DL	0.617	0.330	0.990
Left F3DL	0.549	0.295	0.993
Right F4DL	0.604	0.341	0.992
Left F4DL	0.621	0.353	0.992
Right F5DL	0.616	0.405	0.997
Left F5DL	0.682	0.450	0.996

*MHB* maximum handprint breadth, *F1DL* 1^st^ digit length, *SDL* 2^nd^ digit length, *TDL* 3^rd^ digit length, *F2DL* 4^th^ digit length, *F3DL* 5^th^ digit length

### Significance test

Descriptive statistics (mean, standard deviation) for handprint measurements and the results of paired sample *t* test to analyze the bilateral asymmetry in handprint dimensions for both sexes are presented in Table 2. The mean of all measurements of male subjects was larger than the female subjects. There was no statistically significant bilateral asymmetry in handprint dimensions (*p* > 0.05) in both sexes.

**Table 2:** Descriptive statistics for handprint measurements in male and female participants

Gender	Parameter	Right side		Left side		*t* test	
		Mean	SD	Mean	SD	*t* value	*p* value
Male	MHB	110.64	6.47	110.00	6.48	0.7045	0.4820**
	F1DL	122.77	7.37	122.11	7.41	0.6364	0.5252**
	SDL	175.81	9.19	175.39	9.13	0.3231	0.7469**
	TDL	186.14	9.25	185.48	9.26	0.5020	0.6162**
	F2DL	176.2	8.96	175.65	9.10	0.4295	0.6680**
	F3DL	151.77	8.44	151.28	8.48	0.4110	0.6815**
Female	MHB	98.19	4.08	98.04	4.11	0.2738	0.7845**
	F1DL	114.66	7.40	114.37	7.47	0.2757	0.7831**
	SDL	163.59	6.96	163.39	6.80	0.2185	0.8278**
	TDL	173.01	7.40	172.82	7.47	0.1854	0.8531**
	F2DL	163.24	7.83	163.14	7.83	0.0926	0.9263**
	F3DL	139.90	7.04	139.80	6.94	0.1816	0.9192**

*SD* standard deviation, *MHB* maximum handprint breadth, *F1DL* 1^st^ digit length, *SDL* 2^nd^ digit length, *TDL* 3^rd^ digit length, *F2DL* 4^th^ digit length, *F3DL* 5^th^ digit length

**Not significant (*p* > 0.05)

The mean stature in males and females was 1694.057 ± 62.236 mm and 1566.418 ± 58.554 mm, respectively. The results of the analysis of covariance using GLM revealed the influence of sex on the relationship between stature and handprint measurements (*p* < 0.05). Therefore, the regression models were developed separately by sex.

### Stature estimation from handprint measurements using linear regression analysis

The linear regression equations with the *R*, *R*^2^, SSE, 95% prediction interval, and *p* value to estimate the stature from left- and right-handprint dimensions are presented in Table 3. The investigator or the police does not know whether the handprint was made by a man or a woman. Therefore, to apply the method in real cases where the sex of the subjects is not available from a handprint, the regression equations were developed combining both male and female data. There was a substantial amount of statistical significance (*p* < 0.001) in the correlation coefficients of all the derived regression equations of all handprint parameters of males and females. The values of *R* ranged from + 0.472 to + 0.709 in males, and + 0.333 to + 0.552 in females. Regression equations developed by combining both male and female data revealed a higher value of *R* ranged between + 0.587 and + 0.777. In males, the maximum value of *R* was found between stature and 2^nd^ digit length of right handprint (*R* = + 0.709). On the other hand, in females, the maximum value of *R* was between stature and the right handprint length of right handprint (*R* = + 0.552). However, using the combined data, the right handprint length (*R* = + 0.777) was the most reliable estimator of the stature.

**Table 3:** Linear regression equations to estimate the stature from handprint measurements (in mm)

Sex	Parameter	Side	Equation	***R***	***R***^**2**^	SSE	95% prediction interval	***P*** value
Male	MHB	Right	*S* = 1172.619 + 4.713 MHB	0.490	0.240	54.544	106.906	0.000*
		Left	*S* = 1187.089 + 4.608 MHB	0.480	0.231	54.866	107.537	0.000*
	F1DL	Right	*S* = 1195.433 + 4.060 F1DL	0.481	0.231	54.860	107.526	0.000*
		Left	*S* = 1209.595 + 3.967 F1DL	0.472	0.223	55.138	108.070	0.000*
	SDL	Right	*S* = 849.763 + 4.802 SDL	0.709	0.502	44.141	86.516	0.000*
		Left	*S* = 854.196 + 4.788 SDL	0.703	0.494	44.521	87.261	0.000*
	TDL	Right	*S* = 880.913 + 4.368 TDL	0.649	0.421	47.586	93.269	0.000*
		Left	*S* = 876.747 + 4.406 TDL	0.655	0.429	47.249	92.608	0.000*
	F2DL	Right	*S* = 963.814 + 4.144 F3DL	0.596	0.356	50.213	98.417	0.000*
		Left	*S* = 990.089 + 4.007 F2DL	0.586	0.344	50.674	99.321	0.000*
	F3DL	Right	*S* = 1070.195 + 4.110 F3DL	0.557	0.311	51.939	101.800	0.000*
		Left	*S* = 1083.433 + 4.036 F3DL	0.550	0.303	52.241	102.392	0.000*
Female	MHB	Right	*S* = 1093.306 + 4.817 MHB	0.336	0.113	55.436	108.655	0.001*
		Left	*S* = 1101.291 + 4.743 MHB	0.333	0.111	55.495	108.770	0.001*
	F1DL	Right	*S* = 1275.590 + 2.693 F1DL	0.340	0.116	55.337	108.461	0.001*
		Left	*S* = 1255.961 + 2.714 F1DL	0.346	0.120	55.210	108.212	0.000*
	SDL	Right	*S* = 925.658 + 3.916 SDL	0.446	0.199	52.688	103.268	0.000*
		Left	*S* = 948.659 + 3.781 SDL	0.439	0.193	52.871	103.627	0.000*
	TDL	Right	*S* = 811.028 + 4.365 TDL	0.552	0.305	49.074	96.185	0.000*
		Left	*S* = 824.346 + 4.293 TDL	0.548	0.300	49.237	96.505	0.000*
	F2DL	Right	*S* = 909.671 + 4.023 F2DL	0.537	0.289	49.628	97.271	0.000*
		Left	*S* = 909.628 + 4.038 F2DL	0.540	0.291	49.548	97.114	0.000*
	F3DL	Right	*S* = 937.337 + 4.496 F3DL	0.542	0.293	49.472	96.965	0.000*
		Left	*S* = 956.2276 + 4.364 F3DL	0.517	0.268	50.360	98.706	0.000*
Combined sex	MHB	Right	*S* = 805.626 + 7.897 MHB	0.741	0.549	59.178	115.989	0.000*
		Left	*S* = 801.406 + 7.968 MHB	0.732	0.536	60.017	117.633	0.000*
	F1DL	Right	*S* = 887.060 + 6.260 F1DL	0.599	0.359	70.548	138.274	0.000*
		Left	*S* = 902.111 + 6.158 F1DL	0.587	0.344	71.360	139.866	0.000*
	SDL	Right	*S* = 494.209 + 6.694 SDL	0.767	0.589	56.490	110.720	0.000*
		Left	*S* = 502.096 + 6.660 SDL	0.760	0.578	57.247	112.204	0.000*
	TDL	Right	*S* = 477.699 + 6.418 TDL	0.777	0.603	55.520	108.819	0.000*
		Left	*S* = 475. 834 + 6.444 TDL	0.771	0.595	56.088	109.932	0.000*
	F2DL	Right	*S* = 569.707 + 6.249 F2DL	0.754	0.569	57.860	113.406	0.000*
		Left	*S* = 578.503 + 6.209 F2DL	0.744	0.554	58.841	115.328	0.000*
	F3DL	Right	*S* = 657.345 + 6.671 F3DL	0.742	0.551	59.088	115.812	0.000*
		Left	*S* = 662.986 + 6.646 F3DL	0.729	0.531	60.343	118.272	0.000*

*S* stature, *MHB* maximum handprint breadth, *F1DL* 1^st^ digit length, *SDL* 2^nd^ digit length, *TDL* 3^rd^ digit length, *F2DL* 4^th^ digit length, *F3DL* 5^th^ digit length

*Significant (*p* < 0.001)

The value of *R*^2^ ranged from 0.223 to 0.502 in males, 0.111 to 0.305 in females, and 0.344 to 0.603 in combined data. The SEE in males was varied from ± 44.141 to ± 55.138 mm, in females from ± 49.074 to ± 55.495 mm, and in combined data from ± 55.490 to ± 71.360 mm. The 95% prediction interval ranged from ± 92.608 to ± 107.527 mm in males, ± 96.185 to ± 108.770 mm in females, and ± 108.819 to ± 118.272 mm in the combined sex.

### Stature estimation from handprint measurements using multiple regression analysis

Stature estimation accuracy can be improved by formulating multiple regression equations (Ahemad and Purkait 2011; Ishak et al. 2012b; Paulis 2015). Multiple regression equations are presented in Table 4. All the handprint parameters were combined to formulate the equations that showed a better estimation of stature than the simple linear regression equations. The values of *R* were also improved for multiple regression equations as *R* varied from + 0.731 to + 0.732 in males, + 0.667 to + 0.668 in females, and + 0.815 to + 0.819 in combined sex. The values of *R*^2^ varied from 0.530 to 0.532 in males, 0.362 to 0.373 in females, and 0.664 to 0.671 in combined sex. In males, the SEE was ranged from ± 44.024 to ± 44.107 mm, in females from ± 48.037 to ± 48.526 mm, and in combined sex from ± 51.166 to ± 51.770 mm. The 95% prediction interval ranged from ± 86.117 to ± 86.301 mm in males, ± 93.747 to ± 94.599 mm in females, and ± 100.285 to ± 101.469 mm in combined sex. The regression coefficient of all handprint parameters was statistically significant (*p* < 0.001) with stature.

**Table 4:** Multiple regression equations to estimate the stature from handprint measurements (in mm)

Gender	Equation	***R***	***R***^**2**^	SEE	95% confidence interval	***P*** value
Male	*S* = 857.37 + 1.68 MHB − 1.34 F1DL + 6.46 SDL + 0.41 TDL − 2.40 F2DL + 0.16 F3DL	0.729	0.532	43.937	86.117	0.000*
	*S* = 853.32 + 1.25 MHB − 1.33 F1DL + 5.73 SDL + 2.69 TDL − 4.41 F2DL + 0.89 F3DL	0.728	0.530	44.031	86.301	0.000*
Female	*S* = 751.07 + 1.77 MHB − 1.31 F1DL − 3.67 SDL + 7.04 TDL − 0.46 F2DL + 1.79 F3DL	0.611	0.373	47.830	93.747	0.000*
	*S* = 765.47 + 1.89 MHB − 1.07 F1DL − 3.75 SDL + 6.26 TDL + 1.07 F2DL + 0.68 F3DL	0.601	0.362	48.265	94.599	0.000*
Combined sex	*S* = 460.55 + 3.77 MHB − 1.57 F1DL + 3.34 SDL + 4.10 TDL − 2.60 F2DL + 0.68 F3DL	0.819	0.671	51.166	100.285	0.000*
	*S* = 449.75 + 3.85 MHB − 1.69 F1DL + 3.19 SDL + 4.83 TDL − 2.69 F2DL + 0.21 F3DL	0.815	0.664	51.770	101.469	0.000*

*S* stature, *MHB* maximum handprint breadth, *F1DL* 1^st^ digit length, *SDL* 2^nd^ digit length, *TDL* 3^rd^ digit length, *F2DL* 4^th^ digit length, *F3DL* 5^th^ digit length

*Significant (*p* < 0.001)

## Discussion

In this study, the stature of male subjects (mean 1694.06 ± 62.24 mm) was larger than female subjects (mean 1566.42 ± 58.55 mm). Measurements are larger in males than females were also true for all handprint dimensions (Table 2). A similar type of findings was previously reported by researchers while estimating stature from handprint (Ahemad and Purkait 2011; Ishak et al. 2012b; Krishan et al. 2015; Paulis 2015) and hand (Asadujjaman et al. 2019; Jee and Yun 2015; Pal et al. 2016; Rastogi et al. 2008) measurements.

In our study, no bilateral asymmetry was observed. However, bilateral asymmetry was reported in some previous studies. Ahemad and Purkait (2011) found bilateral asymmetry in handprint width, distal segment of the thumb, and distal and middle segments of the little finger. Ishak et al. (2012b) found bilateral asymmetry in handprint breadth of female subjects. Zulkifly et al. (2018) reported bilateral asymmetry in handprint breadth, index fingers, thumb distal, and index distal phalanges for female subjects.

**Fig. 3:**
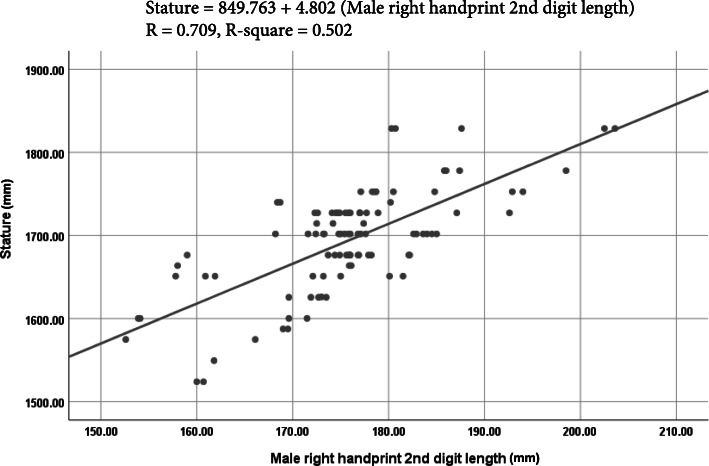
Best fit curve to estimate stature from male right handprint 2^nd^ digit length

**Fig. 4:**
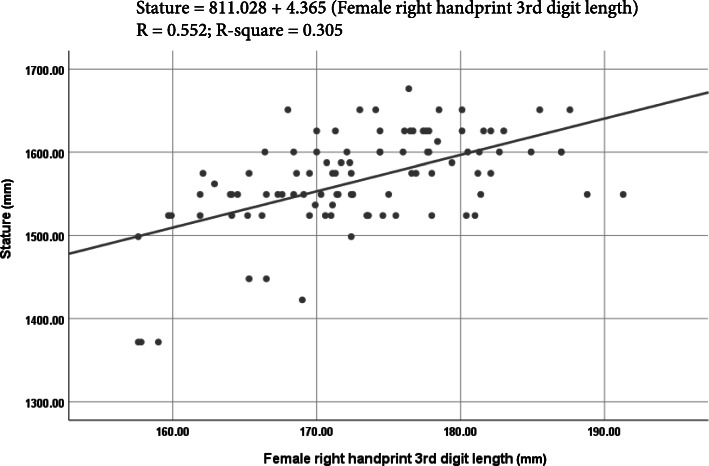
Best fit curve to estimate stature from female right handprint 3^nd^ digit length

**Fig. 5:**
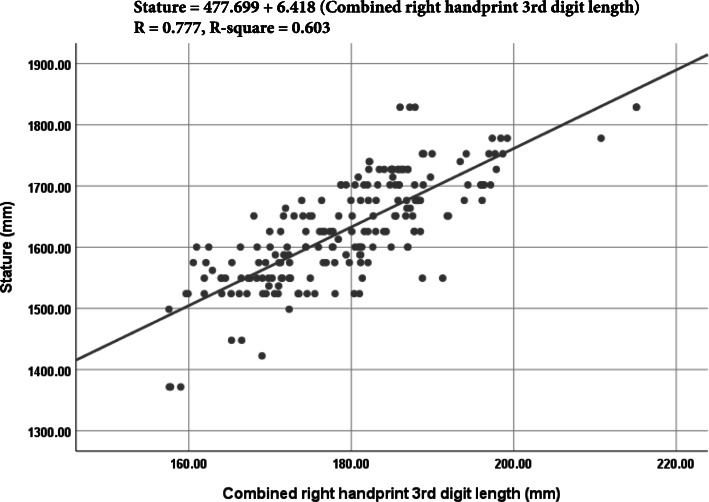
Best fit curve to estimate stature from combined handprint 3^rd^ digit length

From the linear regression analysis (Table 3), it was seen that right handprint 2^nd^ digit length was the most precise single factor to estimate stature from handprint dimensions in males with the lowest value of SSE (± 44.141 mm) and the highest value of *R* (+ 0.709) and *R*^2^ (0.502) in males. In contrast, in females, the right handprint 3^rd^ digit length was reported the highest value *R* (+ 0.552) and *R*^2^ (0.305) with the lowest value of SSE (± 49.074 mm). Therefore, the right handprint 3^rd^ digit length or the right handprint length was the most precise single factor to estimate the stature from handprint dimensions in females. Using the combined data set, the right handprint length was the most reliable single parameter to estimate the stature (*R* = + 0.777, *R*^2^ = 0.603, SEE = ± 55.490 mm). Figures 3, 4, and 5 show the best fit curves to estimate stature using the most reliable single parameter in males, females, and combined data, respectively. A comparative study of the values of the *R* and *R*^2^ between stature and different handprint measurements found among various populations is shown in Tables 5 and 6, respectively. Similar to this present study, all the previous studies (Ahemad and Purkait 2011; Ishak et al. 2012b; Paulis 2015; Zulkifly et al. 2018) found a positive correlation between the handprint measurements and the stature. Handprint length (i.e., the 3^rd^ digit length in this study) was the only common handprint measurement between this study and previous studies. Therefore, the comparison was presented with respect to hand length. In the study of Paulis (2015) done on the Egyptian population, right hand length of males (*R* = 0.519, *R*^2^ = 0.270, SSE = ± 45.40 mm) and females (*R* = 0.298, *R*^2^ = 0.089, SSE = ± 53.80 mm) were more dependable. In a study of central Indian male population by Ahemad and Purkait (2011), hand length (*R* = 0.558, *R*^2^ = 0.312, SSE = ± 46.35 mm) was the most reliable parameter for estimating stature. In another study of the Western Australian population, Ishak et al. (2012b) reported that the right handprint length in males (*R* = 0.640, *R*^2^ = 0.410, SSE = ± 54.20 mm) and left handprint length of females (*R* = 0.650, *R*^2^ = 0.420, SSE = ± 54.60 mm) were the most reliable parameters. Similarly, the right handprint length in both males (*R* = 0.680, *R*^2^ = 0.460, SSE = ± 55.50 mm) and females (*R* = 0.640, *R*^2^ = 0.410, SSE = ± 46.70 mm) were also noted by Zulkifly et al. (2018) as most dependable parameters for estimating the human height.

**Table 5:** Comparison of correlation coefficient (*R*) among different studies

Measurements	Present study						Paulis (2015)		Ahemad and Purkait (2011)	Ishak et al. (2012b)				Zulkifly et al. (2018)			
	Male		Female		Combined sex		Male	Female	Male	Male		Female		Male		Female	
	Right	Left	Right	Left	Right	Left	Right	Right	Combined side	Right	Left	Right	Left	Right	Left	Right	Left
MHB	0.490	0.480	0.336	0.333	0.741	0.732											
F1DL	0.481	0.472	0.340	0.346	0.599	0.587											
SDL	0.709	0.703	0.446	0.439	0.767	0.760											
TDL	0.649	0.655	0.552	0.548	0.777	0.771	0.519	0.298	0.558	0.640	0.640	0.650	0.650	0.680	0.650	0.640	0.600
F2DL	0.596	0.586	0.537	0.540	0.754	0.744											
F3DL	0.557	0.550	0.542	0.517	0.742	0.729											

*MHB* maximum handprint breadth, *F1DL* 1^st^ digit length, *SDL* 2^nd^ digit length, *TDL* 3^rd^ digit length, *F2DL* 4^th^ digit length, *F3DL* 5^th^ digit length

**Table 6:** Comparison of coefficient of determination (*R*^*2*^) among different studies

Measurements	Present study						Paulis (2015)		Ahemad and Purkait (2011)	Ishak et al. (2012b)				Zulkifly et al. (2018)			
	Male		Female		Combined sex		Male	Female	Male	Male		Female		Male		Female	
	Right	Left	Right	Left	Right	Left	Right	Right	Combined side	Right	Left	Right	Left	Right	Left	Right	Left
MHB	0.240	0.231	0.113	0.111	0.549	0.536											
F1DL	0.231	0.223	0.116	0.120	0.359	0.344											
SDL	0.502	0.494	0.199	0.193	0.589	0.578											
TDL	0.421	0.429	0.305	0.300	0.603	0.595	0.269	0.089	0.312	0.410	0.410	0.420	0.420	0.460	0.420	0.410	0.360
F2DL	0.356	0.344	0.289	0.291	0.569	0.554											
F3DL	0.311	0.303	0.293	0.268	0.551	0.531											

*MHB* maximum handprint breadth, *F1DL* 1^st^ digit length, *SDL* 2^nd^ digit length, *TDL* 3^rd^ digit length, *F2DL* 4^th^ digit length, *F3DL* 5^th^ digit length

Different handprint dimensions were used by different researchers as the independent variable of the linear regression equations for estimating the stature (Table 7). Paulis (2015) developed regression equations using handprint breadth, handprint length, and phalangeal length, whereas the SSE was between ± 45.4 to ± 58.9 mm in males, and ± 53.8 to ± 66.8 mm in females. Moreover, Ahemad and Purkait (2011) used handprint length, handprint width, palm length, thumb distal, complete thumb, index distal, (index distal + middle), complete index, middle distal, (middle distal + middle), complete middle, ring distal, (ring distal + middle), complete ring, little distal (little distal + middle), and complete little measurements for developing regression equations. In their study, the SSE was ranged from ± 46.35 to ± 54.95 mm in males. Furthermore, Ishak et al. (2012b) derived equations form handprint length, handprint breadth, palm print length, and thumb, index, ring, middle fingerprint length and reported the SSE in between ± 54.2 and ± 61.3 mm in males, and ± 54.6 and ± 64.0 mm in females. Further, Zulkifly et al. (2018) used handprint breadth; handprint length; handprint thumb, index, middle, ring, little finger length; phalangeal length of thumb distal, thumb proximal, index distal, index middle, index proximal, middle distal, middle medial, middle proximal, ring distal, ring middle, ring proximal, little distal, little middle; and little proximal dimensions for developing regression equations. The SSE values were ranged from ± 54.2 to ± 75.5 mm in males, and ± 46.7 to ± 59.9 mm in females. In this present study, the regression equations were derived using handprint breadth, maximum handprint breadth, 1st digit length, 2nd digit length, 3rd digit length, 4th digit length, and 5th digit length. In comparison, our study revealed lower values of SSE (± 44.141 to ± 55.138 mm in males, ± 49.074 to ± 55.495 mm in females, and ± 55.490 to ± 71.360 mm in combined data) from some of the previous studies (Ishak et al. 2012b; Paulis 2015; Zulkifly et al. 2018).

**Table 7:** Comparison of studies dealing with the stature estimation from the hand measurements using linear regression

Study	Sample	Ages	Population	Parameters	Standard error
Paulis (2015)	100 males and 91 females	18–67	Egyptian	Handprint breadth; Handprint length; Phalangeal length	45.4–58.9 mm in males and 53.8–66.8 mm in females
Ahemad and Purkait (2011)	503 males	18–35	Indian	Handprint length, Handprint width, palm length, thumb distal, complete thumb, index distal, index distal + middle, complete index, middle distal, middle distal + middle, complete middle, ring distal, ring distal + middle, complete ring, little distal, little distal + middle, complete little	46.35–54.95 mm in males
Ishak et al. (2012b)	91 males and 110 females	16–68	Western Australian	Handprint length; Handprint breadth; Palm print length; Thumb, index, ring, middle fingerprint length;	54.2–61.3 mm in males and 54.6–64.0 mm in females
Zulkifly et al. (2018)	50 males and 52 females	18–60	Iban	Handprint breadth; Handprint length; Handprint thumb, index, middle, ring, little finger length; Phalangeal length of thumb distal, thumb proximal, index distal, index middle, index proximal, middle distal, middle medial, middle proximal, ring distal, ring middle, ring proximal, little distal, little middle, little proximal	54.2–75.5 mm in males and 46.7–59.9 mm in females
Present study	100 males and 100 females	18–30	Bangladeshi	Handprint Breadth; Maximum Handprint Breadth; 1st Digit Length; 2nd Digit Length; 3rd Digit Length 4th Digit Length; 5th Digit Length	44.141–55.138 mm in males, 49.074–55.495 mm in females, and 55.49–71.36 mm in combined data

The multiple regression equations were developed for both hands and for both sexes, to improve the accuracy of estimation (Table 4). When all the parameters were considered for stature estimation, the accuracy was increased for both sexes and both hands as the values of *R* have increased and the values of SSE have decreased in every case. The values of SSE ranged from ± 43.937 to ± 44.031 mm in males, and ± 47.830 to ± 48.265 mm in females, which was lower than the simple linear regression models. The largest value of *R* (+ 0.729) and the lowest value of SSE (± 43.937 mm) was found in the right hand of males. In contrast, the lowest value of *R* (+ 0.601) and the highest value of SSE (± 48.26 mm) was found in the left handprint of males. Therefore, the right handprint dimensions in males were more reliable, and the left handprint dimensions in females were less reliable for the estimation of stature. However, using combined sex data, multiple regression models showed greater reliability in stature estimation. The right handprint measurements showed the highest reliability with a value of *R* = + 0.819, *R*^2^ = 0.671, and SEE = ± 51.166 mm in combined sex. These findings are similar to the past research where the estimation accuracy was improved by using multiple regression analysis (Ishak et al. 2012b; Paulis 2015; Zulkifly et al. 2018).

### Application of handprint in forensic practice

When there are no suspects comparing, estimating stature from the handprints found at a crime scene can help the law enforcement agencies to narrow down the pool of suspects. The past studies revealed that handprint measurements could be used in the estimation of stature. Several studies were done on various populations, such as Indian (Krishan et al. 2015), Egyptian (Paulis 2015), Western Australian (Ishak et al. 2012b), and Iban (Zulkifly et al. 2018) population.

Handprints have been used by law enforcement authorities in many cases to identify the criminals even when they already have several suspects (Handprint left at “massacre” scene 2002; Suspect linked to bloody handprint in 1986 killing pleads not guilty 2019; Forensic expert testifies about bloody hand prints found in apartment as Berry murder trial continues 2019). Handprints may be found in various forms such as on a flat surface, or on soil or mud. In developing countries (e.g., Bangladesh, Pakistan, India), clash or fight between two parties in the open field is common. In case of clash or fight between two parties, handprint may find on soil or mud. For instance, in 2015, 10 injured in Brahmanbaria clash (2015) where people fight in the open field. Even in this Covid 19 period, in Brahmanbaria, a clash between villagers happened in an open ground (Villagers keep fighting amid coronavirus 2020). In such cases, where when people fight in the field, the injured even can die owing to fighting, where handprint may be found in the soil and that may be used to identify the criminals or victims.

Various method of handprint acquisition was used by previous researchers. Sharma and Kapoor (2001) narrated a method of estimating stature from fingertip length and fingerprint length using inked impressions. Further, Jasuja and Singh (2004) collected handprint measurements by taking an inked impression of hands and estimated stature from handprint length and breadth. Moreover, Ahemad and Purkait (2011) collected handprints by taking an inked impression and only studied men subjects only. Zulkifly et al. (2018) also used ink impression of hands for collecting handprint dimensions. Furthermore, Ishak et al. (2012b) collected scanned images of hands and from the printed copies of these images hand and finger dimensions were manually measured. Beside this, Paulis (2015) automated the handprint collection process and used a computer to measure the handprint dimensions from scanned images of hands.

**Fig. 6:**
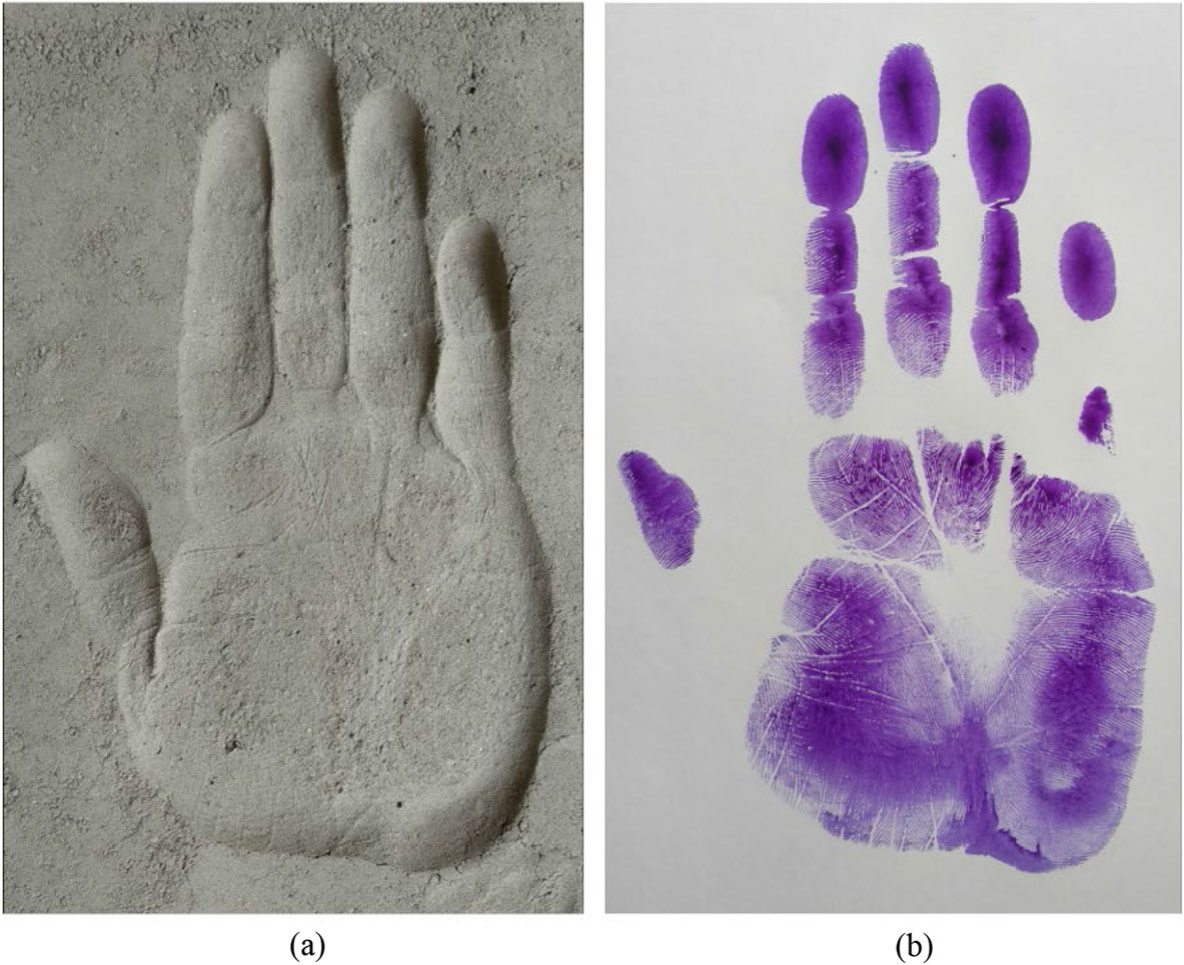
**a** Handprint on soil. **b** Handprint on flat surface

In this study, the handprint dimensions were collected by taking hand impressions on powdered soil, which was a new method and not examined in the literature. However, handprint may be found on a flat surface. Therefore, the difference between the handprint on soil and the flat surface has been examined in this study. Figure 6 shows the hand impression on the soil and the flat surface of the same participant at the same hand pressure. Ink impression was used to take the handprint on the flat surface (Fig. 6b). The difference between handprint measurements on soil and flat surface is presented in Table 8. It was seen that the handprint impression on soil was larger than the handprints on the flat surface. When the hand impression was taken on the flat surface, full hand area did not touch the flat surface. On the other hand, at the same pressure when hand impression was taken on soil, hand penetrates a little bit into the soil owing to the softness. Therefore, handprint dimensions were larger on soil than the flat surface. The ratio of handprint measurements on soil and flat surface is useful to identify the individuals, whether the hand impression is found on the soil or the flat surface.

**Table 8:** Difference of handprint measurement on soil and flat surface

Sex	Side	Parameter	Mean handprint on soil	Mean handprint on flat surface	Handprint ratio (soil/flat surface)
Male	Right	MHB	99.83	94.96	1.05
		F1DL	117.15	113.48	1.03
		SDL	167.55	162.70	1.03
		TDL	180.32	174.36	1.03
		F2DL	170.54	164.85	1.03
		F3DL	147.95	143.04	1.03
	Left	MHB	102.38	96.39	1.06
		F1DL	118.23	113.13	1.05
		SDL	169.55	165.71	1.02
		TDL	181.36	177.80	1.02
		F2DL	173.67	167.62	1.04
		F3DL	150.78	144.77	1.04
Female	Right	MHB	94.00	87.50	1.07
		F1DL	104.50	100.00	1.05
		SDL	151.00	149.00	1.01
		TDL	165.00	159.50	1.03
		F2DL	156.00	152.50	1.02
		F3DL	135.50	129.00	1.05
	Left	MHB	93.50	88.00	1.06
		F1DL	102.00	101.50	1.00
		SDL	153.50	150.00	1.02
		TDL	167.00	164.00	1.02
		F2DL	154.50	153.00	1.01
		F3DL	137.50	131.00	1.05

*MHB* maximum handprint breadth, *F1DL* 1^st^ digit length, *SDL* 2^nd^ digit length, *TDL* 3^rd^ digit length, *F2DL* 4^th^ digit length, *F3DL* 5^th^ digit length

## Conclusion

This study reported the application of handprint measurements in stature estimation in Bangladeshi adults. The stature can be reliably estimated from the handprint dimensions. The 2^nd^ digit length of the right handprint in males, and the handprint length in females were the most reliable single parameter for the estimation of stature. Using combined sex data, the handprint length was the most reliable single parameter to estimate the stature. The estimation accuracy was increased in the case of multiple regression analysis. This study uses the hand impression on soil which is not reported in previous studies. Therefore, this study examines the measurement difference between the handprint on soil and flat surface. Consequently, this study is useful to find the stature of unknown individuals, whether the hand impression is found on a flat surface or soil. For forensic, medicolegal, and crime investigation purposes, the new standard to estimate the stature has a great impact in Bangladesh. The pool of suspects can be narrowed down form the handprints found at a crime scene and thus can be cross-matched with other pieces of evidence. The age of subjects taken in this study of the Bangladeshi population ranged between 18 and 30 years. In the future, new studies can be done on subjects for other different age ranged people of Bangladesh.

## Data Availability

Owing to the confidentiality, data of this study will not be shared for public access.

## Abbreviations

SD: Standard deviation
MHB: Maximum handprint breadth
F1DL: 1^st^ digit length
SDL: 2^nd^ digit length
TDL: 3^rd^ digit length
F2DL: 4^th^ digit length
F3DL: 5^th^ digit length
